# MicroRNA Profiling Implies New Markers of Chemoresistance of Triple-Negative Breast Cancer

**DOI:** 10.1371/journal.pone.0096228

**Published:** 2014-05-02

**Authors:** Mao Ouyang, Yongxin Li, Sheng Ye, Jieyi Ma, Liming Lu, Weiming Lv, Guangqi Chang, Xiaoxi Li, Qing Li, Shenming Wang, Wenjian Wang

**Affiliations:** 1 Laboratory of Department of Surgery, First Affiliated Hospital, Sun Yat-sen University, Guangzhou, Guangdong, People's Republic of China; 2 Center for Cellular and Structural Biology, Guangzhou, Guangdong, People's Republic of China; 3 Department of Clinical Laboratory, First Affiliated Hospital, Sun Yat-sen University, Guangzhou, Guangdong, People's Republic of China; 4 Department of Medical Oncology, First Affiliated Hospital, Sun Yat-sen University, Guangzhou, Guangdong, People's Republic of China; 5 Department of Medical Statistics and Epidemiology, Sun Yat-sen University, Guangzhou, Guangdong, People's Republic of China; 6 Department of Vascular, Thyroid and Breast Surgery, First Affiliated Hospital, Sun Yat-sen University, Guangzhou, Guangdong, People's Republic of China; Rutgers - New Jersey Medical School, United States of America

## Abstract

**Objective:**

Triple-negative breast cancer (TNBC) patients with truly chemosensitive disease still represent a minority among all TNBC patients. The aim of the present study is to identify microRNAs (miRNAs) that correlate with TNBC chemoresistance.

**Methods:**

In this study, we conducted miRNAs profile comparison between triple-negative breast cancer (TNBCs) and normal breast tissues by microRNA array. Quantitative real-time PCR (qRT-PCR) was utilized to confirm the specific deregulated miRNAs change trend. We used starBase 2.1 and GOrilla to predict the potential targets of the specific miRNAs. Cells viability and apoptosis assays were employed to determine the effect of alteration of the specific miRNAs in TNBC cells on the chemosensitivity.

**Results:**

We identified 11 specific deregulated miRNAs, including 5 up-regulated miRNAs (miR-155-5p, miR-21-3p, miR-181a-5p, miR-181b-5p, and miR-183-5p) and 6 down-regulated miRNAs (miR-10b-5p, miR-451a, miR-125b-5p, miR-31-5p, miR-195-5p and miR-130a-3p). Thereafter, this result was confirmed by qRT-PCR. We predicted the potential targets of the candidate miRNAs and found that they are involved in cancer-associated pathways. For the first time, we found that miR-130a-3p and miR-451a were down-regulated in TNBC. 9 of the 11 specific deregulated miRNAs were found to be associated with chemoresistance. In vitro assays, we found that up-regulation of either miR-130a-3p or miR-451a in MDA-MB-231 cells significantly changed the cells sensitivity to doxorubicin. The results suggest that TNBC chemotherapy might be affected by a cluster of miRNAs.

**Conclusion:**

The abnormal expression miRNAs in TNBC are mainly chemoresistance related. This might be part of reason that TNBC likely to evade from chemotherapy resulting in early relapse and high risk of death. To alter their expression status might be a potential therapeutic strategy to improve the outcome of chemotherapy for TNBC patients.

## Introduction

Primary breast cancer is usually classified into different categories based on the gene expression profile, phenotype and susceptibility to therapy. TNBC is a kind of invasive carcinoma of primary breast cancer that lacking expression of estrogen receptor (ER), progesterone receptor (PR) and human epidermal growth factor receptor 2 (HER2), which accounts for about 12–17% of all breast cancers including patients with stage I–IV breast cancer. TNBC is usually associated with higher cell proliferation and more chance of recurrence, invasion and metastasis [1]. Since the expressions of ER, PR and HER2/neu conventionally determine the therapeutic response and general disease prognosis of primary breast cancer, methods for the treatment of TNBC patients are still limited in clinical. TNBC is not sensitive to endocrine therapy and HER2 targeted therapy, so chemotherapy is vital for the treatment of TNBC. TNBC patients with truly chemosensitive disease still represent a minority among all TNBC patients [2], [3]. And the subgroup as a whole with residual disease have worse disease free and overall survival [4]. To identify the subgroup of TNBC patients with chemosensitive disease and predict biomarkers for personalizing use of chemotherapy is of great value to improve the prognosis of TNBC patients.

miRNAs are non-coding RNAs that consist of 21 to 22 nucleotides and have critical role in tumorigenesis and progression by controlling cell proliferation, differentiation, invasion, migration and apoptosis via regulating the stability or inhibiting the translation of their target mRNAs [5]. miR-122 was downregulated in breast cancer and overexpression of miR-122 could inhibit cell proliferation and tumorigenesis of breast cancer by targeting IGF1R [6]. miR-340 could inhibit breast cancer cell migration, as well as invasion. And endogenous miR-340 expression was down-regulated in the more aggressive breast cancer cell lines, especially in TNBC cell lines [7]. These results indicated that miRNA could play a role as tumor-suppressor. MiR-221 was significantly increased in breast cancer cells and overexpression of miR-221 promoted cell proliferation and invasion by targeting 14-3-3ζ and uPAR [8], [9]. The result suggested that miRNA could also function as an oncomiR. Accumulating studies suggested some miRNAs were correlated with chemoresistance. Besides promotion of growth, migration and invasion, overexpression of miR-205 could enhance the chemoresistance of non-small cell lung cancer (NSCLC) cells by targeting PTEN [10]. Overexpression of miR-181a enhanced the chemoresistance to cisplatin by targeting PRKCD in human cervical squamous cell carcinoma [11]. Therefore, we supposed that abnormal expression of miRNAs might contribute to either development or chemotherapy efficiency of TNBC.

This study aimed to identify tumor-specific miRNAs which might involve in TNBC carcinogenesis and chemotherapy by miRNA profile comparison between TNBCs and normal breast tissues. The results were clarified with bioinformatics analysis, literature review and qRT-PCR. Our results might help to dig out potential chemoresistance-related biomarkers or treatment targets for TNBC.

## Materials and Methods

### Samples and patients

The study was approved by the ethic committee of the First Affiliated Hospital of Sun Yat-sen University. Written informed consent for use of biomaterials was obtained from all patients. Fresh TNBC tissues and their adjacent normal tissues were obtained from 15 TNBC patients who underwent surgical resection between January 2011 and October 2011 in the Department of Breast Surgery, the First Affiliated Hospital of Sun Yat-sen University. All patients had received anthracyclines or taxanes-based adjuvant chemotherapy after surgery. Patients' ages ranged from 34 to 57years (mean age, 43.2 years). All cases had been confirmed by Hematoxylin-Eosin staining and immunohistochemical detection for ER, PR and HER-2, respectively. 3 pairs of fresh TNBC tissues and their adjacent normal tissues were used to identify miRNAs expression signatures. The other 12 pairs were used to conduct qRT-PCR detection.

### miRNA array experiment and data process

Total RNA was harvested using Trizol (Invitrogen) and miRNAeasy mini kit (QIAGEN) according to manufacturer's instructions. RNA quantity measurement was performed by NanoDrop 1000. Then the RNA samples were labeled with miRCURY Hy3/Hy5 Power labeling kit and hybridized on the miRCURY LNA Array 16.0. Following the washing steps, the slides were scanned using the Axon GenePix 4000B microarray scanner. Scanned images were then imported into GenePix Pro 6.0 software (Axon) for grid alignment and data extraction. Replicated miRNAs were averaged and miRNAs that intensities ≥50 in all samples were chosen for calculating normalization factor. Expressed data were normalized using the median normalization. After normalization, significant differentially expressed miRNAs were identified through Volcano Plot filtering. Finally, hierarchical clustering was performed to show distinguishable miRNA expression profiling among samples. We selected specific miRNA signatures according to their fold change, bioinformatics analysis (gene ontology, pathway and network analysis) and literature review.

### Targets prediction

To predict the potential targets of the specific deregulated miRNAs, we utilized starBase 2.1 which is a public platform for decoding miRNA-targets, combining of five prediction programs (TargetScan, PicTar, miRanda, PITA and RNA22) [12]. Known experimentally validated miRNA targets were downloaded from miRTarBase 3.5. Enrichment analysis of the specific miRNAs targets was performed using the web-based tool GOrilla. StarBase database was also used for enrichment analysis for the KEGG pathways of target genes of the selected specific miRNAs. Cytoscape 3.0.2 was utilized to construct the possible functional network of the selected miRNAs [13].

### Quantitative real-time polymerase chain reaction

Total RNA was prepared from the 12 pairs of fresh TNBC and normal breast samples with Trizol reagent (Invitrogen) and the concentration of total RNA was quantitated by measuring the absorbance at 260 nm. qRT-PCR for miRNAs was performed with cDNA generated from 1 µg of total RNA, using a SYBR Premix EX TaqTM II kit (Takara, Dalian, China), according to the manufacturer's instructions. All primers were designed by Takara. U6 was used for miRNA normalizations. Then the reverse transcription (RT) reaction mixture (25 ul) was subjected to qRT-PCR analyses using CFX96 (Bio-Rad Laboratories, Hercules, CA) according to the manufacturer's instructions. Fluorescent signals were normalized to U6 and the threshold cycle (Ct) was set within the exponential phase of the PCR. The relative expression levels were calculated and quantified by using the 2^−ΔΔCt^ method after normalization. qRT-PCR was performed in triplicate.

### Cell culture

The normal breast epithelial cell line MCF 10A was cultured in mammary epithelial cell growth medium (Clonetics) supplemented with 100 ng/ml cholera toxin. The TNBC cell lines, MDA-MB-231, BT-549 and Hs 578T were cultured in Dulbecco's modified Eagle's medium (Gibco) supplemented with 10% fetal bovine serum. All cell lines were purchased from American Type Culture Collection (ATCC) and cultured at 37°C in a humidified atmosphere with 5% (v/v) CO2 in air.

### Cell transfection

MDA-MB-231 cells were seeded in 96 well plate (10000 cells/well) or 6 well plate (100000 cells/well) and allowed to adhere overnight. Then, cells were transfected with 100 nM mimc of miR-130a-3p or miR-451a, respectively. 100 nM mimc of a non-specific miRNA was used as negative control. Tansfection was performed with Lipofectamine 2000 (Invitrogen) according to the manufacture's instruction. miRNA mimics used in this study were designed by and purchased from Ribobio (Ribobio Co., China).

### Cells viability and apoptosis assays

After transfection, MDA-MB-231 cells were treated with or without 0.2 ug/ml doxorubicin for 48 h, and harvested. The vehicle control contained 0.9% NaCl (pH 7.2). Thereafter, cells viability was tested by using cell counting kit-8 (Beyotime, China) according to the instruction. The absorbance at 450 nm of each well was read on a spectrophotometer. At the same time, cells apoptotic rate was determined by using Annexin V-FITC and PI staining flow cytometry kit (KeyGEN BioTECH, China) according to manufacture's instruction. Briefly, cells in different transfection groups were harvested and washed with PBS for twice. After that, cells were resuspended in 500 µl binding buffer provided by the kit. 5 µl Annexin V and 5 µl propidium iodide (PI) were added to the cells and then incubated at room temperature for 15 minutes in dark. Cells apoptotic rate was then tested by flow cytometry within 1 h.

### Statistical analysis

All data were expressed as mean ± SD. Statistical analysis was performed with One-way ANOVA followed by Dunnett's Multiple Comparison Test. A probability value of 0.05 was accepted as statistically significant. All data were processed using SPSS 11.5 (SPSS, Chicago, IL).

## Results

### miRNA expression signatures differentiate between TNBC and normal tissues

**Figure 1:**
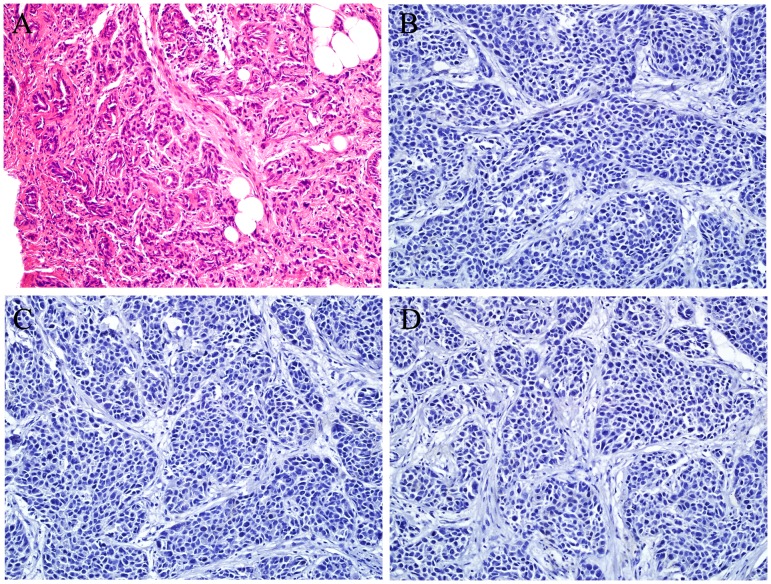
Microphotographs (×200) of a representative TNBC case. A: Hematoxylin-eosin staining. B–D: Immunohistochemical detection for ER, PR and HER-2, respectively. This case is negative for ER, PR and HER-2 (triple negative).

**Figure 2:**
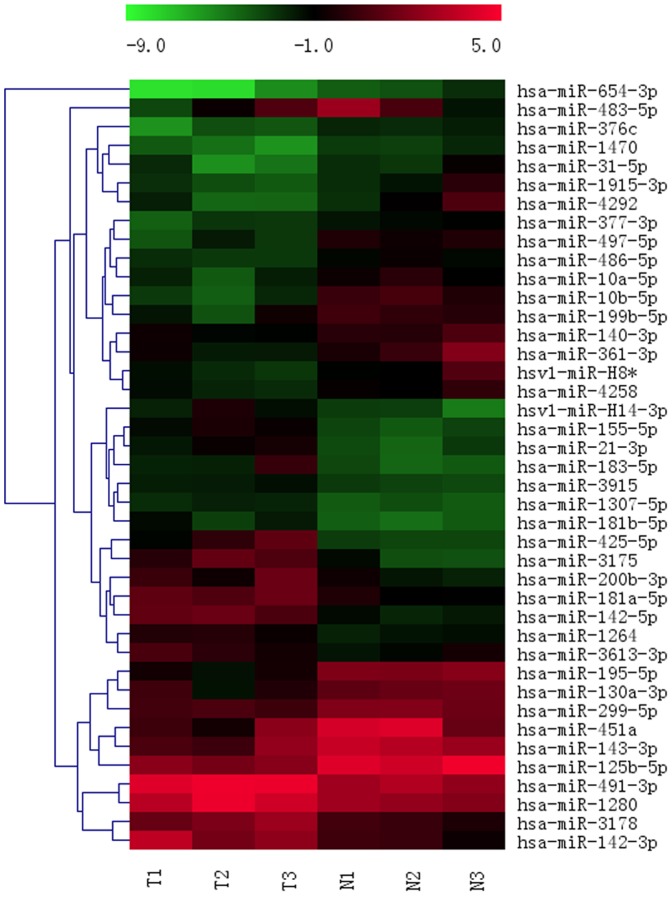
miRNA differential expression in TNBC versus normal breast tissues. Hierarchical clustering of the 41 miRNAs with a significantly different expression. Rows, individual miRNAs; columns, individual tissue samples. Pseudocolors represent transcript levels above, equal to, and below the mean (red, black, and green, respectively). The scale represents the intensity of miRNA expression (log2 scale ranges between −9 and 5).

TNBC cases were identified by Hematoxylin-Eosin staining and routine immunohistochemistry against ER, PR and HER-2, respectively (Figure 1). We examined the expression levels of totally 1513 miRNAs in 3 paired TNBCs and adjacent normal tissues by microarray (Table S1). After normalization and removing the miRNAs with missing value in any tissue, 597 miRNAs were used to perform hierarchical clustering. Finally, 41 significantly different expression miRNAs were identified (Figure 2).

18 of them were significantly up-regulated and the other 23 were down-regulated. Some of them have been reported in previous studies. For example, the significantly over-expression of miR-21 and under-expression of miR-122 were found in TNBCs by qPCR [10]. Eventually, we chose 11 specific deregulated miRNAs according to their fold change, bioinformatics analysis and literature review about their potential role in tumorigenesis and treatment.

As shown in Figure 2, the levels of miR-155-5p, miR-21-3p, miR-181a-5p, miR-181b-5p, miR-183-5p were up-regulated, while the levels of miR-10b-5p, miR-451a, miR-125b-5p, miR-31-5p, miR-195-5p, miR-130a-3p were down-regulated in TNBC group versus matched peritumoral counterparts.

### Expression status of the 11 selected miRNAs detected by qRT-PCR

**Figure 3:**
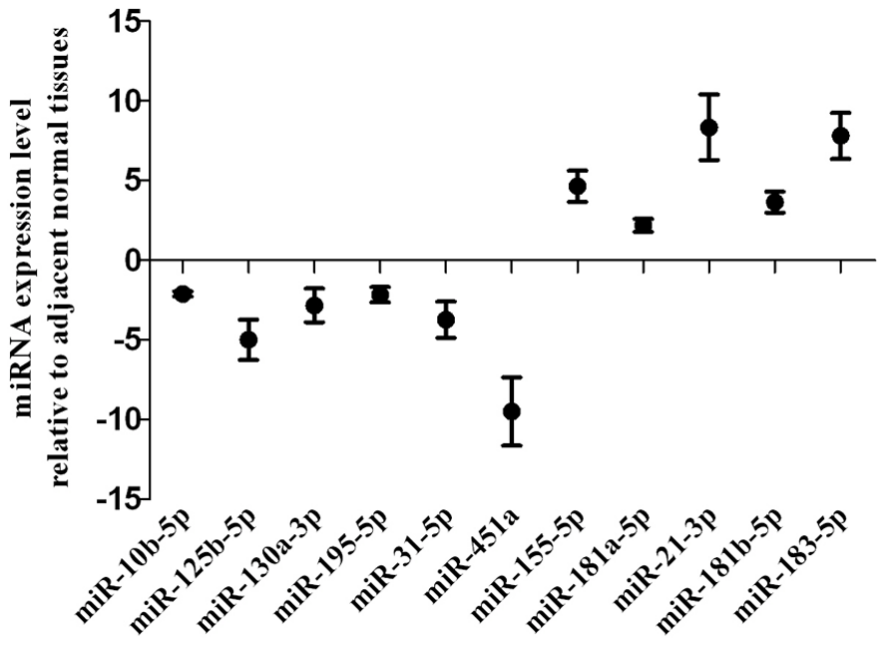
qRT-PCR validation of miRNAs microarray results in TNBCs. Relative expression of miRNAs in TNBCs compared with their adjacent normal tissues by qRT-PCR. miR-10b-5p, miR-451a, miR-125b-5p, miR-31-5p, miR-195-5p, miR-130a-3p were down-regulated in cancer samples, whereas miR-155-5p, miR-21-3p, miR-181a-5p, miR-181b-5p and miR-183-5p were up-regulated. Dots, normalized ratio of miRNA expression values (TNBC/adjacent normal tissues).

To confirm the microarray results, we utilized qRT-PCR to detect expression status of the 11 specific selected miRNAs in other 12 pairs of TNBCs and their adjacent normal tissues. qRT-PCR results showed that miRNA change trend was well consistent with the microarray (Figure 3).

### Predicted functional networks of the deregulated miRNAs

To elucidate the potential role of the specific selected 11 miRNAs in TNBC, targets analyses were performed with starBase platform. A large number of genes were predicted as targets of the selected miRNAs, including many well-known genes that are deregulated in breast cancer, such as CCND1, BCL2, E2F3 and PTEN.

**Figure 4:**
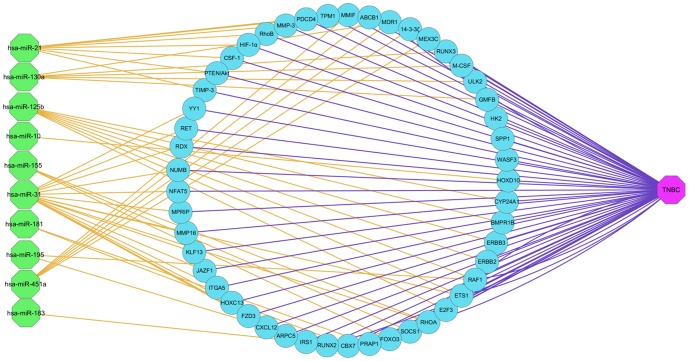
Network among the specific deregulated miRNAs and their predicted targets in this study.

To probe functional networks of candidate miRNA target genes, we utilized miR Pathway from starBase to analyze the possible pathways that contain the putative targets genes of the selected miRNAs. We found that the set of genes regulated by the 11 deregulated miRNAs was enriched for proteins that have key roles in various pathways (Figure 4), such as PTEN/Akt, MAPK, RhoA, FOXO3 and PDCD4 genes, which are causatively deregulated in cancer diseases. These genes mainly involved in cellar proliferation, differentiation, migration, invasion and apoptosis, etc. After further analysis (combining pathway prediction with literature review), we found miR-155, miR-21, miR-181a, miR-181b, miR-10b, miR-451a, miR-125b, miR-31 and miR-130a-3p were all involved in chemoresistance related pathways (Table 1).

**Table 1:** Chemoresistance-related miRNAs of the 11 selected miRNAs.

MicroRNAs	Location	Confirmed Targets	Alteration	Drug Resistance	Tissue Type	Authors
**miR-155**	21q21.3	FOXO3a	↑	cisplatin	TNBC	Ling N, et al.
**miR-21**	17q23.1	PDCD4, Fas-L	↑	cisplatin	nasopharyngeal carcinoma	Yang G, et al.
		PTEN	↑	VM-26	prostate cancer	Shi G, et al.
		MSH2	↑	cisplatin	TNBC	Yu Y, et al.
**miR-181a**	9q33.3	PRKCD	↑	cisplatin	cervical squamous cell carcinoma	hen Y, et al.
		Twist1	↑	cisplatin	tongue squamous cell carcinoma	Liu M, et al.
**miR-10b**	2q31.1	BCL2L11	↑	5-fluorouracil	colorectal cancer	Nishida N, et al.
**miR-451**	17q11.2	ABCB1	↓	irinotecan	colon cancer	Bitarte N, et al.
		MDR1	↓	doxorubicin	breast cancer	Kovalchuk O, et al.
**miR-125b**	11q24.1	E2F3	↑	5-FU	breast cancer	Wang H, et al.
		Bak-1	↑	paclitaxel	breast cancer	Zhou M, et al.
**miR-31**	9p21.3	MET	↓	taxane	ovarian cancer	Mitamura T, et al.
**miR-130a**	11q12.1	MDR1	↓	paclitaxel	ovarian cancer	Sorrentino A, et al.
		RUNX3	↓	cisplatin	hepatocellular carcinoma	Xu N, et al.
		M-CSF	↓	paclitaxel	ovarian cancer	Sorrentino A, et al.
**miR-181b**	1q32.1	Bcl2	↑	gemcitabine	pancreatic ductal adenocarcinoma	Cai, B, et al.
		Bcl2	↑	temozolomide	glioma	Li P, et al.
**miR-183**	7q32.2	Ezrin	↓		osteosarcoma	Zhu J, et al.
**miR-195**	17p13.1	GLUT3	↓		bladder cancer	Fei X, et al.

### Overexpression of miR-130a-3p or miR-451a in MDA-MB-231 cells led to chemosensitivity to doxorubicin

**Figure 5:**
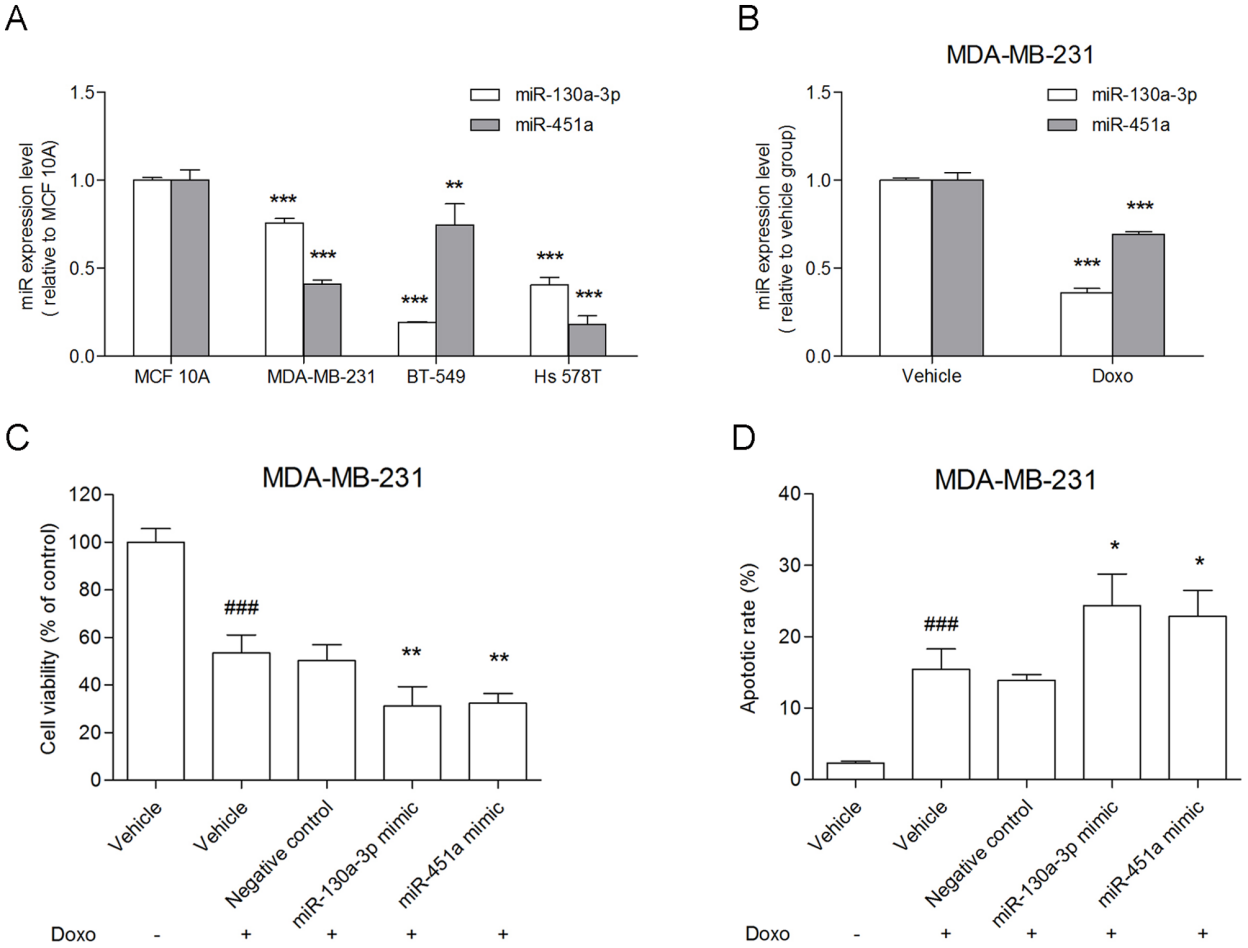
Up-regulation of miR-130a-3p or miR-451a significantly changed MDA-MB-231 cells sensitivity to doxorubicin. A: Expression levels of miR-130a-3p or miR-451a were evaluated in different cell lines by qRT-PCR (** *P*<0.01; *** *P*<0.001, compared to MCF 10A). B: During doxorubicin treatment, miR-130a-3p or miR-451a were significantly down-regulated in MDA-MB-231 cells, respectively (*** *P*<0.001, compared to vehicle control). C–D: Cells viability and cells apoptotic rate were analyzed by the cell counting kit-8 assay and flow cytometry, respectively (### *P*<0.001, compared to vehicle control; * *P*<0.05, ** *P*<0.01, compared to Doxo group). All the results were obtained from three independent experiments. Doxo, doxorubicin.

Firstly, we did comparisons of expression of both miR-130a-3p and miR-451a between TNBC cells, MDA-MB-231, BT-549 and Hs 578T, and normal breast epithelial cells, MCF 10A. The expression levels of either miR-130a-3p or miR-451a in all the TNBC cells were significantly lower than that of normal breast epithelial cells (Figure 5A). Either miR-130a-3p or miR-451a in MDA-MB-231 cells were significantly down-regulated after treated with 0.2 ug/ml doxorubicin for 48 h (Figure 5B). To confirm that miR-130a-3p and miR-451a are linked to TNBC chemoresistance, we conducted cell viability and apoptosis assays using MDA-MB-231, which was treated by doxorubicin. We found that overexpression of either miR-130a-3p or miR-451a in MDA-MB-231 cells could significantly decrease cells viability, and increase cells apoptotic rate compared with the doxorubicin group (Figures 5C and 5D).

## Discussion

In the present study, we identified a global expression pattern of miRNAs in TNBC. Finally, 11 miRNAs closely related with TNBC were selected according to their fold change, bioinformatics analysis and literature review about their potential role in tumorigenesis and treatment. We confirmed the expression pattern of the 11 miRNAs by qRT-PCR. The expression levels of miR-155-5p, miR-21-3p, miR-181a-5p, miR-181b-5p and miR-183-5p were up-regulated in TNBC tissues, while the levels of miR-10b-5p, miR-451a, miR-125b-5p, miR-31-5p, and miR-195-5p and miR-130a-3p were down-regulated. Our study is consistent with previous studies [11], [14]. Among the selected miRNAs, there was none report of miR-451a in TNBCs. No study referred miR-130a-3p to either TNBC or breast cancer. We utilized starBase to predict the potential targets of the selected miRNAs. Cancer-associated pathways of the miRNAs had been drafted by starBase analysis as well. The main signaling pathways include PTEN/Akt, MAPK, MDR1, RhoA, FOXO3 and PDCD4 pathway, which play vital roles in regulating tumor cellular proliferation, migration, invasion, apoptosis, etc. Notably, except miR-183 and miR-195, all other miRNAs are chemoresistance- related. Whether this is part of reason that TNBC likely to evade from chemotherapy resulting in early relapse and high risk of death remains elusive.

miR-130a was an important oncomiR by repressing mitogen-activated protein kinase (MAPK) signaling pathway, which could promote vascular endothelial cell proliferation and angiogenesis in tumor [15]. Besides MAPK, we predicted the potential target signaling pathways of miR-130a including ULK2 and GMFB. Function analysis showed that they were related with autophagy and proliferation, respectively. Increasing evidence showed that miR-130a played a vital role in cancer chemoresistance. Overexpression of miR-130a could lead to drug resistance while downregulation of miR-130a could overcome cisplatin resistance by targeting MDR1/P-glycoprotein in SKOV3/CIS cells [16]. A similar study found that miR-130a levels were over-expressed in cisplatin resistance hepatocellular carcinoma cell lines. Up-regulation of miR-130a directly inhibited expression of tumor-suppressor gene RUNX3 leading to activation of Wnt/β-catenin signaling and sequent drug resistance [17]. These results suggested that overexpression of miR-130a was related with chemoresistance. A conflicting report showed that miR-130a was down-regulated in chemoresistant ovarian cancer cell lines. Down-regulation of miR-130a could enhance chemoresistance by targeting M-CSF, which could induce drug-resistant cell phenotype [18]. The difference might be due to the genetics of the evaluated cell lines and diversity in methods used. The function of miR-130a in TNBC is still uncertain. In our study, we found miR-130a-3p was down-regulated in either TNBC cell lines or clinical samples. Expression of miR-130a-3p in MDA-MB-231 cells decreased after treatment with doxorubicin. Up-regulation of miR-130a-3p in MDA-MB-231 cells significantly increased the cells sensitivity to doxorubicin. The results indicate that miR-130a-3p might be a new marker in TNBC chemoresistance.

miR-451 plays critical role in cancer cellular growth, migration, invasion in various of cancers by targeting MMIF, PI3k/Akt, RAB14, LKB1/AMPK [19]–[23]. Furthermore, our prediction indicated that 14-3-3ζ and MEX3C might be potential targets of miR-451a. Studies showed that 14-3-3ζ played important role in cellular proliferation and migration through enhancing MAPK/c-Jun signaling [24]. MEX3C played a critical role in cellular growth by up-regulating insulin-like growth factor 1 (IGF1) expression [25]. Recently studies indicated that miR-451 was a chemoresistance biomarker. miR-451 was down-regulated in chemoresistance colon cancer cells. Up-regulation of miR-451 could cause a decrease in tumorigenicity and chemoresistance to irinotecan of colonspheres by directly inhibiting direct target macrophage migration inhibitory factor (MMIF), and finally down-regulating expression of cyclooxygenase-2 (COX-2) [19]. In addition, transfection of the doxorubicin-resistant MCF-7 breast cancer cells with miR-451 resulted in a decrease of MDR1 gene product, p-glycoprotein (P-gp), and increased sensitivity of MCF-7 cells to doxorubicin [26]. Same result had been found in non-small cell lung cancer cell line (A549). Up-regulation of miR-451 could significantly increase the sensitivity of A549 cell to cisplatin by increasing DDP-induced apoptosis [27]. These results suggest that correction of altered expression of miR-451 may be a new therapeutic strategy aiming to overcome chemoresistance. However, little is known about the role of miR-451a in TNBC chemoresistance. We found miR-451a expression was down-regulated in TNBC. Treatment with doxorubicin decreased the expression of miR-451a in MDA-MB-231 cells. Transfection of MDA-MB-231 cells with miR-451a significantly increased the cells sensitivity to doxorubicin. The results suggest that miR-451a might act as a new marker in TNBC chemotherapy efficacy.

miR-21, miR-155, miR-181a, miR-181b, miR-183, miR-10b, miR-125b, miR-31 and miR-195 had been widely studied in cancer diseases including TNBC. Their roles include regulation of tumor cellular proliferation, migration, invasion and apoptosis. From literature review, we found that most of these miRNAs are associated with chemoresistance, except miR-195 and miR-183.

Over-expression of miR-21 could contribute to resistance to DNA-damaging chemotherapy agents via MSH2 in TNBC cells [28]. Up-regulation of miR-21 could enhance chemoresistance in nasopharyngeal carcinoma cells by targeting PDCD4 and Fas-L [29]. Enforced expression of miR-21 could also induce chemoresistance in glioblastoma multiforme cells by targeting LRRFIP1 [30]. Overexpression of miR-155 in breast cancer cell lines (including TNBC cell line) increased the cells chemoresistance and survival by targeting FOXO3a [31]. A similar study had been reported in colon cancer HT29 cells for miR-155 [32]. Enforced expression of miR-181a could decrease chemo-sensitivity to cisplatin in cervical cancer cells through PRKCD [33]. Up-regulation of miR-181a reversed chemoresistance by targeting Twist1 in tongue squamous cell carcinoma [34]. Overexpression of miR-181b reduced chemoresistance to temozolomide in glioma stem cells by targeting Bcl-2 [35]. A similar result of miR-181b had been observed in pancreatic ductal adenocarcinoma cells [36]. Down-regulation of miR-125b sensitized breast cancer cells to chemotherapy by targeting E2F3 [37]. Overexpression of miR-125b enhanced resistance of ovarian cancer cells to cisplatin by targeting Bak1 [38]. miRNA-31 reduction induced taxane resistance in ovarian cancer cells through increase of MET [39]. Overexpression of miR-10b induced resistance to 5-fluorouracil in colorectal cancer cells by targeting BCL2L11 [40].

Overall, most of the 11 deregulated miRNAs are associated with chemoresistance, indicating that TNBC chemotherapy might be affected by a cluster of miRNAs.

In conclusion, miRNAs profiling identified 11 specific deregulated miRNAs in TNBCs. 9 of the deregulated miRNAs were found to be associated with chemoresistance, which had been confirmed by previous studies. To our best knowledge, this is the first report that miR-130a-3p and miR-451a are down-regulated in TNBC. To alter the expression status of miR-130a-3p or miR-451a in MDA-MB-231 cells significantly changed the cells sensitivity to doxorubicin. The results suggest that TNBC chemoresistance might be associated with a cluster of deregulated miRNAs. Furthermore, to alter expression status of the 9 deregulated miRNAs might be a potential therapeutic option to improve chemotherapy outcome of TNBCs.

## Supporting Information

Table S1 **miRNAs profile comparison between TNBC and normal breast tissues.**
(XLS)
